# Circulating miRNAs in Women with Polycystic Ovary Syndrome: A Longitudinal Cohort Study

**DOI:** 10.3390/cells12070983

**Published:** 2023-03-23

**Authors:** Pernille B. Udesen, Anja E. Sørensen, Rikke Svendsen, Nanna L. S. Frisk, Anne L. Hess, Mubeena Aziz, Marie Louise M. Wissing, Anne Lis M. Englund, Louise T. Dalgaard

**Affiliations:** 1 Fertility Clinic, Department of Gynecology and Obstetrics, Zealand University Hospital, Lykkebækvej 14, 4600 Koege, Denmark; 2 Department of Science and Environment, Universitetsvej 1, 4000 Roskilde, Denmark; 3 Department of Nutrition, Exercise and Sports, Faculty of Science, University of Copenhagen, Rolighedsvej 26, 1958 Frederiksberg C, Denmark; 4 Department of Gynecology and Obstetrics, Amager/Hvidovre Hospital, Kettegaards Allé 30, 2650 Hvidovre, Denmark

**Keywords:** biomarker, metabolic syndrome, microRNA, PCOS, polycystic ovary syndrome, circulation, ovary, non-coding RNA, clinical trial

## Abstract

Background: Women with polycystic ovary syndrome (PCOS) often change their metabolic profile over time to decrease levels of androgens while often gaining a propensity for the development of the metabolic syndrome. Recent discoveries indicate that microRNAs (miRNAs) play a role in the development of PCOS and constitute potential biomarkers for PCOS. We aimed to identify miRNAs associated with the development of an impaired metabolic profile in women with PCOS, in a follow-up study, compared with women without PCOS. Methods and materials: Clinical measurements of PCOS status and metabolic disease were obtained twice 6 years apart in a cohort of 46 women with PCOS and nine controls. All participants were evaluated for degree of metabolic disease (hypertension, dyslipidemia, central obesity, and impaired glucose tolerance). MiRNA levels were measured using Taqman^®^ Array cards of 96 pre-selected miRNAs associated with PCOS and/or metabolic disease. Results: Women with PCOS decreased their levels of androgens during follow-up. Twenty-six of the miRNAs were significantly changed in circulation in women with PCOS during the follow-up, and twenty-four of them had decreased, while levels did not change in the control group. Four miRNAs were significantly different at baseline between healthy controls and women with PCOS; miR-103-3p, miR-139-5p, miR-28-3p, and miR-376a-3p, which were decreased in PCOS. After follow-up, miR-28-3p, miR-139-5p, and miR-376a-3p increased in PCOS women to the levels observed in healthy controls. Of these, miR-139-5p correlated with total testosterone levels (rho = 0.50, p_adj_ = 0.013), while miR-376-3p correlated significantly with the waist-hip ratio at follow-up (rho = 0.43, p_adj_ = 0.01). Predicted targets of miR-103-3p, miR-139-5p, miR-28-3p, and miR-376a-3p were enriched in pathways associated with Insulin/IGF signaling, interleukin signaling, the GNRH receptor pathways, and other signaling pathways. MiRNAs altered during follow-up in PCOS patients were enriched in pathways related to immune regulation, gonadotropin-releasing hormone signaling, tyrosine kinase signaling, and WNT signaling. Conclusions: These studies indicate that miRNAs associated with PCOS and androgen metabolism overall decrease during a 6-year follow-up, reflecting the phenotypic change in PCOS individuals towards a less hyperandrogenic profile.

## 1. Introduction

Polycystic Ovary Syndrome (PCOS), defined by hyperandrogenism, oligo- or anovulation, and polycystic ovary morphology according to the Rotterdam criteria [1], is also characterized by insulin resistance, which is a part of the pathogenesis [2]. The etiology of PCOS is not known in detail but is mainly caused by ovarian and endocrine disturbances, which in younger women primarily manifests as reproductive symptoms including infertility, hyperandrogenism, irregular menstrual cycles, polycystic ovarian morphology, and an increased risk of pregnancy complications if pregnancy occurs [1]. Over time, these symptoms become less prominent, and metabolic disturbances, which are closely related to the pathophysiology of PCOS, become more pronounced. Women with PCOS have an increased risk of developing metabolic syndrome (MetS) and have a four times higher risk of presenting with type 2 diabetes (T2D) [3,4].

MicroRNA (miRNA) are small, non-coding RNA molecules that regulate gene expression by post-transcriptional inhibition of targeted mRNAs [5]. MiRNAs bind to their target mRNA and either inhibit translation or promote their degradation. As present in all body compartments and in circulation, miRNAs are suitable as biomarkers [6]. Furthermore, miRNAs are protected from degradation by Ribonucleases because they are contained in extracellular vesicles, bound in protein complexes or lipoproteins [6]. Studies have shown an altered expression of miRNAs in insulin-sensitive tissues from obese or overweight individuals and patients with T2D, suggesting a potential role of these small RNA molecules in the complications associated with MetS and T2D [7,8].

Another quality of miRNAs as biomarkers is the ability to reflect the changes in clinical disease stages. This has been demonstrated in weight-loss studies and studies of miRNA levels during treatment with insulin sensitizers and bariatric surgery [9,10,11].

While the correlation between metabolic disease and levels of specific miRNAs is well described [12,13], the correlation between miRNAs and reproductive hormones and PCOS is still debated. Several studies have investigated the miRNA profile in women with PCOS [14,15], investigated miRNA biomarkers in serum or plasma [16,17,18,19,20], or searched for miRNAs in follicular fluid [16,21,22] to unravel the biological mechanisms behind hyperandrogenism and anovulation of PCOS. The results of these studies are, however, of great variability, perhaps because of the complexity and heterogeneity of PCOS, and only a few studies have been able to validate previous findings, although some miRNAs have been reported in more than one study. Several studies have reported miR-93 levels altered in both adipose tissue, serum, and granulosa cells in women with PCOS [23,24,25]. Likewise, other research groups have shown that miR-21 is altered in serum, granulosa cells, and follicular fluid [26,27]. Several recent reviews have covered this active field of research [14,15,28]. However, none of these studies addressed the change in miRNA levels over time in PCOS patients, and it is not known how the changes in clinical markers and reproductive hormones of women with PCOS are reflected in the miRNA profile over time; knowledge which is essential in order for miRNA to serve as biomarkers. Thus, with this longitudinal study, we aim to assess selected miRNAs in PCOS women before assisted reproductive therapy and after an average of 6 years of follow-up (FU) to investigate the relationship between the miRNA profile and metabolic changes over time.

## 2. Materials and Methods

### 2.1. Study Design and Participants

**Figure 1:**
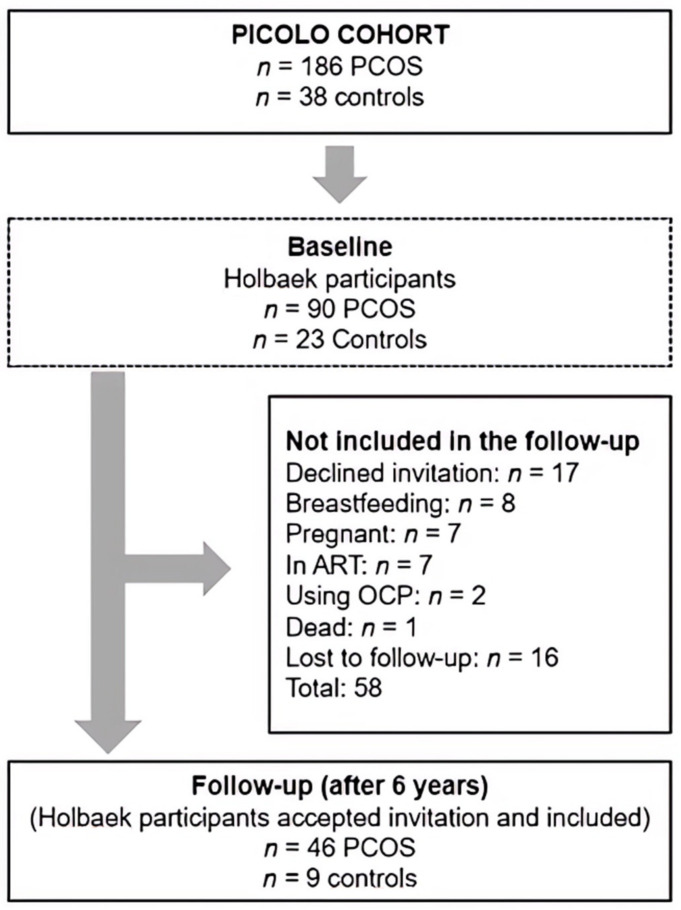
Flowchart of included women with PCOS and controls at BL and at FU.

The present study consists of a FU study of 46 women with PCOS and nine controls. Women were diagnosed according to the Rotterdam 2003 consensus criteria [29]. This study was based on the PICOLO cohort of 186 women with PCOS and 38 healthy controls recruited between 2010 and 2013 from three Danish hospitals: Herlev, Hvidovre, and Holbaek Hospital [30]. A flow chart of the participants is shown in Figure 1. Descriptions of the PICOLO cohort can be found in [21,31,32]. The control group and the PCOS group were not matched at baseline (BL) but were similar regarding BMI and age in the FU study.

In the FU study, all participants of the PICOLO study from Holbaek Hospital were invited for FU re-examination at Holbaek Fertility Clinic 6 years after the first visit (Baseline; BL). Exclusion criteria: Oral contraceptives within 8 weeks of examination, endocrine disease, endometriosis, premature ovarian insufficiency, breastfeeding, and pregnancy (Figure 1). Serums from BL and FU were used for miRNA analysis. This study followed the Declaration of Helsinki II, was approved by the Danish Data Protection Agency (REG-31-2016) and the Danish Scientific Ethical committee of region Zealand (Journal no. SJ-525), and was registered at Clinicaltrials.gov (NCT03142633). All subjects gave informed written consent prior to inclusion.

### 2.2. Anthropometric and Clinical Characteristics at FU

Clinical examination at FU included anthropometric measurements (height, weight, blood pressure (BP)), vaginal ultrasound, blood sampling, and an oral glucose tolerance test (OGTT).

An OGTT was performed after an overnight fast (>8 h). We collected venous blood samples and measured glucose, serum insulin, and C-peptide at −5, 0, 30, and 120 min after a 75 g glucose load. HOMA-IR was calculated as (fasting insulin (µU/mL) * fasting glucose (mmol/l))/22.5 [33]. Clinical hirsutism was evaluated with Ferriman-Gallwey score (FG-score). Total testosterone (T) was measured using liquid chromatography mass spectrometry (Xevo TQD, Waters, Milford, MA, USA). Sex hormone binding globulin (SHBG), plasma C-peptide, serum insulin, and dehydroepiandrosterone sulfate (DHEAS) were measured on ADVIA Centaur^®^ XP (Siemens Healthcare, Oxford, UK). Free T was calculated from total T and sex hormone binding globulin (SHBG) described by Vermeulen et al. [34]. Plasma total cholesterol, high-density lipoprotein (HDL) cholesterol and triglycerides, luteinizing hormone (LH), follicle-stimulating hormone (FSH), and androstenedione were measured according to standard laboratory procedures at Holbaek Hospital.

### 2.3. Serum MiRNA Isolation and Analysis

MiRNA analyses were conducted using serum from venous blood samples collected in a procoagulant drying tube. Serum and cellular fractions were separated by centrifugation at 2000 rpm for 20 min. Serum was carefully removed, leaving 0.5 mL in order to avoid disturbance of the interface. The serum was stored at −80 °C until analysis. Total RNA was extracted from serum with TriReagent LS (Sigma-Aldrich Denmark, Copenhagen, Denmark), according to manufacturer’s protocol. RNA concentration and purity were determined using NanoDrop ND-1000 (ThermoFisher Scientific, Hvidovre, Denmark).

RNA was reverse transcribed using a 96 miRNA custom-designed TaqMan Reverse Transcription Kit (ThermoFisher Scientific, Hvidovre, Denmark) according to the manufacturer’s protocol. The RT-product was pre-amplified with TaqMan^®^ PreAmp Master Mix and miRNA PreAmp Custom Primer Pools. MiRNA levels were measured by quantitative PCR using human TaqMan low density array (TLDA) cards containing 96 miRNAs (ThermoFisher Scientific, Hvidovre, Denmark) (Table S1). The custom array card was designed with miRNAs from the literature associated with PCOS, metabolic syndrome, insulin resistance or features of the metabolic syndrome, and in some cases circulating miRNAs associated with ovarian cancer along with three miRNAs selected based on an in-house study [32]. Expression data were obtained using the ViiA 7 real-time PCR system and analyzed using QuantStudio software.

Amplification plots were manually inspected, and assays with absent or poor amplification or low fluorescent intensity were excluded for further analysis. Cycle threshold (Ct) >32 was considered undetectable following manufacturer’s guidelines and excluded from the analysis. A miRNA had to be present in a minimum of ten subjects to be included in further analysis steps. Thirteen (*n* = 13) miRNA at FU and nine (*n* = 9) miRNA at BL did not fulfill these criteria. Data were normalized against the geometric mean of two references, the endogenous small nuclear U6 and miR-484 using the delta-delta Ct method and log-transformed for further analysis.

### 2.4. Statistical Analysis

All data were analyzed with Statistical Packages for Social Sciences (SPSS, vers. 27, IBM, USA) and GraphPad Prism (Version 9.1.2, GraphPad Inc., La Jolla, CA, USA). All data are presented as means (SD) or medians (IQR) if not normally distributed. Categorical variables are presented as numbers (percentages). All data were tested with the Shapiro-Wilk test for normal distribution. The following variables did not follow normal distributions and were log-transformed: Free T, total T, SHBG, FG-score, FSH, LH, androstenedione, total cholesterol, HDL- and LDL-cholesterol, triglycerides, glucose, insulin, C-peptide, HOMA-IR and miRNAs. For statistical analysis, grouped data were compared with a students’ *t*-test for unpaired and paired samples or one-way ANOVA with a Tukey post-hoc test. Visualization of data was made using RStudio (v. 1.3.1093, RStudio, PBC Boston, MA, USA, http://www.rstudio.com (accessed on 20 February 2023)). Heatmaps were made with the R-package ‘pheatmap’ (vers. 1.0.12 [35]). Correlations were assessed with Pearson’s test and visualized using the R-package ‘corrplot’ (vers. 0.89 [36]). Binary logistic regression was used to predict dichotomous outcomes using correction for BMI and age as well in order to create receiver operating characteristic curves (ROC) analysis. The discriminating power of ROC analysis is presented in the form of an AUC (R package ´cutpointr´, vers. 1.1.1 [37]). Missing data were handled with pairwise deletion. A *p*-value of <0.05 was considered statistically significant.

## 3. Results

### 3.1. Clinical Phenotype at FU in Control and PCOS Subjects

The median FU time was 6.1 years (range 4.0–7.1 years). The women with and without PCOS were comparable both at BL and at FU (Table 1), except for a higher w/h-ratio at BL in the PCOS group (*p* = 0.03). BMI increased during the study period in both groups (PCOS: *p* = 0.02, controls: *p* = 0.02), the w/h-ratio increased in the control group only (*p* = 0.03), and the diastolic BP decreased in the PCOS only (*p* = 0.02) (Table 1). The PCOS group presented, as expected, with significantly higher measures of androgen parameters (total T, free T, androstenedione, and FG-score) at BL. These differences persisted at FU, except for free T. Comparing BL with FU, the PCOS group demonstrated a significant decrease in all androgen parameters, whereas the control group only showed a significant decrease in total T and androstenedione (Table 1).

Regarding metabolic parameters, the groups were comparable at BL and at FU. However, when comparing BL with FU using paired samples analysis, the PCOS group significantly increased their serum-insulin, C-peptide and HOMA-IR over time. The fasting glucose declined during this study in both groups but only significantly in the PCOS group. Control and PCOS women were comparable with regard to the success of assisted reproductive therapy (ART) and the degree of smoking and alcohol consumption, while metformin use was frequent (Table 1).

### 3.2. Longitudinal Changes in MiRNA Levels

The longitudinal design enabled an evaluation of the changes in miRNA levels over time. Out of the 96 tested miRNAs, levels of thirty miRNAs were significantly different in PCOS women from BL to FU (Table 2). The majority (22) of these miRNAs were decreased in levels. These miRNAs were present in at minimum half of the PCOS women and with absolute fold changes greater than two. None of the tested miRNAs changed over time within the control women (Table S2).

### 3.3. MiRNA Profile Associated with a Shift in Metabolic Profile

**Figure 2:**
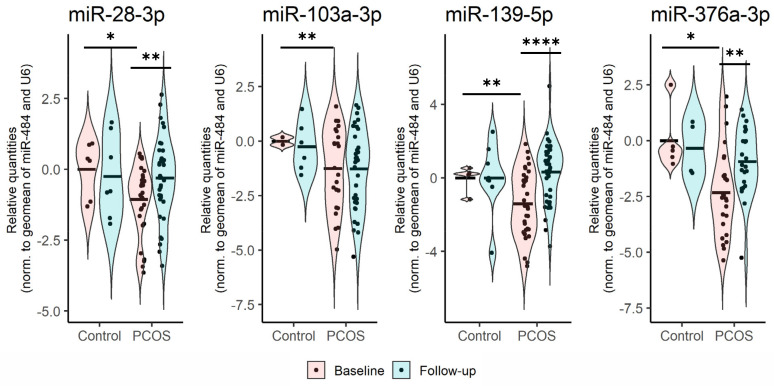
Relative expression of miRNAs which were significantly different at BL between healthy controls and women with PCOS. Levels of the individual miRNAs were normalized against the geometric mean of miR-484 and U6. The comparison of BL or FU levels was made with an independent student *t*-test, while a paired *t*-test was used for comparing BL vs. FU levels. *: *p* < 0.05, **: *p* < 0.001, **** *p* < 0.00001.

When comparing the levels of the 96 selected miRNAs, lower levels of four miRNAs (miR-28-3p (*p* < 0.045), miR-103a-3p (*p* < 0.009), miR-139-5p (*p* < 0.005) and miR-376a-3p (*p* < 0.017)) were observed in women with PCOS already at BL compared with controls (Figure 2). At FU, women with PCOS presented with miRNA levels similar to those observed in the controls. Subsequently, these miRNAs changed significantly over time within the PCOS group (miR-28-3p (*p* < 0.003), miR-139-5p (*p* < 0.00001), and miR-376a-3p (*p* < 0.005)), except for miR-103-3p (Figure 2). Interestingly, we found no differences in the 96 pre-selected miRNAs, including the aforementioned four miRNAs at FU, when comparing the two groups.

### 3.4. BL Circulating MiRNAs Are Associated with PCOS

**Figure 3:**
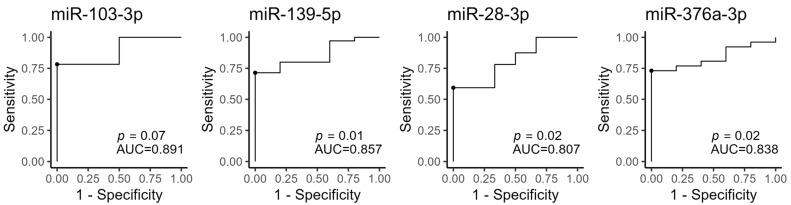
ROC curves of the four miRNAs measured at BL. The optimal cut-off point (Youden index) is marked with a black circle. AUC: Area under the curve. miR-103-3p: *n* = 25; miR-139-5p: *n* = 40; miR-28-3p: *n* = 38, miR-376a-3p: *n* = 31.

Given the heterogeneous nature of PCOS, we investigated whether miRNAs, which were significantly different at BL, could be used to discriminate between women with PCOS and healthy controls using ROC-curve analysis. Using logistic regression to include age and BMI at BL, both circulating miR-139-5p (area under the curve (AUC) = 0.857, *p* = 0.01), miR-376a-3p (AUC = 0.838, *p* = 0.02) and miR-28-3p (AUC = 0.807, *p* = 0.02) showed high discriminatory power with a specificity of a 100% based on an optimal threshold value (the Youden index) (Figure 3). Sensitivities ranged from 78% to 59% (order from highest to lowest; miR-376a-3p, -139-5p, and -28-5p). When combining free T with age and BMI at BL, the ROC-curve analysis yields a lower discriminatory power (AUC = 0.788, *p* = 0.01, Figure S1); the discriminatory power of single miRNAs is therefore comparable to, or even better than, free T.

### 3.5. Androgen Levels and LH/FSH Ratio Correlate with MiRNA Levels at BL

We investigated which of the clinical and biochemical markers correlated with the four miRNAs at either BL or FU within the PCOS women (Table 3 and Supplementary Figure S2). At BL, both miR-139-5p (ρ = 0.50, *p* = 0.002) and miR-28-3p (ρ = 0.37, *p* = 0.043) correlated with total T and miR-139-5p also correlates with free T (ρ = 0.47, *p* = 0.005) within PCOS women (Table 3). Likewise, LH/FSH ratio correlated positively with both miR-139-5p (ρ = 0.36, *p* = 0.038) and miR-28-3p (ρ = 0.40, *p* = 0.028) (Table 3). The women with PCOS did not alter their waist-hip ratio significantly from BL to FU. However, at FU, their miR-376a-3p levels had increased and presented with a positive correlation be the two (ρ = 0.43, *p* = 0.042). Of interest, total T still correlated with miR-28-3p at FU (ρ = 0.40, *p* = 0.016). Both the association between miR-376a-3p and waist-hip ratio and between total T and miR-28-3p remained significant upon controlling for the effects of age and BMI at the individual time points (Table 3).

### 3.6. Pathway Enrichment Analysis for the Four MiRNAs Different between PCOS and Control Women during FU

**Figure 4:**
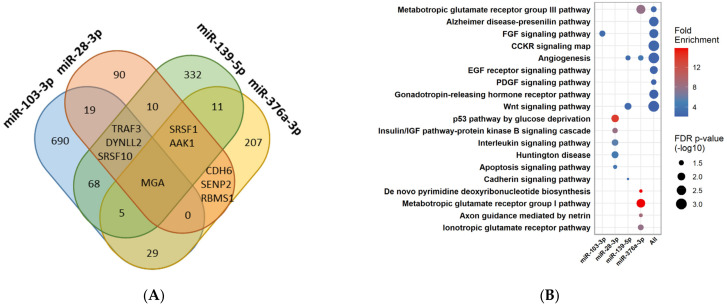
Overview of predicted targets and pathway enrichment analysis of each of the four miRNAs, which change differentially over time in PCOS women compared with controls, as well as a combination hereof. (**A**) Venn diagram of predicted targets identified through TargetScan v.7.2. (human). (**B**) Enrichment analysis of predicted targets. The color of the circles corresponds to the degree of positive fold enrichment, while the size of the circle corresponds to the false discovery rate (FDR) adjusted *p*-values.

To gain insight into possible miRNA functionality, predicted target genes of the four miRNAs were used to construct a pathway enrichment analysis (Figure 4). A total of 1470 unique predicted targets was identified (Figure 4A) with limited overlaps between predicted targets of all four miRNAs. The 3′ untranslated region of the MAX gene-associated protein (MGA) was common among all of the four miRNAs. Genes targeted by miR-103-3p were enriched within the fibroblast growth factor (FGF) signaling pathway-a pathway relevant for both proliferation of granulosa cells as well as steroidogenesis (Figure 4B).

One of the most highly enriched pathways was the p53 pathway by glucose deprivation, which contains genes predicted to be targeted by mIR-28-3p. Furthermore, genes belonging to the insulin/IGF pathway and apoptosis signaling pathway were predicted to be targeted by miR-28-3p (Figure 4B). Three pathways associated with either angiogenesis, Wnt signaling, or Cadherin signaling were enriched by genes targeted by miR-139-5p. Lastly, miR-376-3p targeted genes within either pyrimidine de novo biosynthesis, glutamate receptor pathways, or genes associated with axon guidance mediated by netrin (Figure 4B). Furthermore, using a combination of all of the predicted targets demonstrated, besides the already mentioned pathways, that pathways associated with hormonal regulation and insulin resistance were also among the enriched ones (Figure 4B).

### 3.7. Pathway Enrichment Analysis for the MiRNAs Significantly Changed in PCOS Women during FU

**Figure 5:**
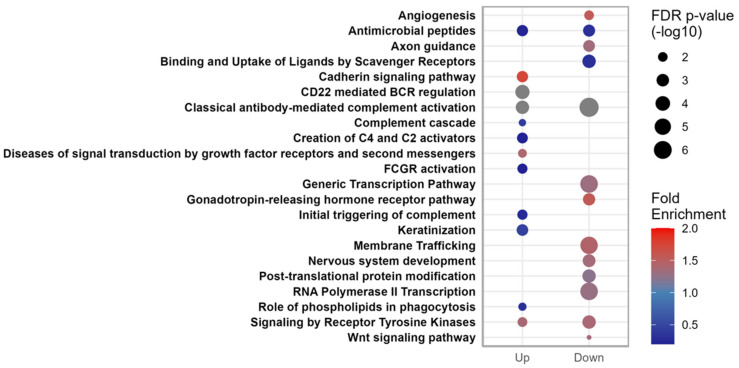
Overview of predicted pathway enrichments of the mRNA targets of the miRNAs significantly changed during FU, either upregulated or downregulated in PCOS patients. The color of the circles corresponds to the degree of positive fold enrichment, while the size of the circle corresponds to the false discovery rate (FDR) adjusted *p*-values.

To obtain information regarding possible miRNA functional actions, we analyzed the enriched pathways of predicted target genes of either the upregulated or the down-regulated miRNAs in PCOS patients during the 6-yr FU (Figure 5). A general observation was that immune-regulation and inflammatory pathways were depleted among the target mRNAs of miRNAs increased in PCOS patients during the FU, while cadherin signaling, growth factor receptor signaling, and tyrosine kinase receptor signaling were enriched among target mRNAs of miRNAs increased in PCOS patients. MiRNAs decreased in PCOS patients during FU had mRNA targets enriched in membrane trafficking, nervous system development, angiogenesis, and post-translational modifications, while these miRNAs had mRNA targets depleted in pathways of antimicrobial peptides and binding and uptake of scavenger receptors.

## 4. Discussion

With this longitudinal FU study, we aimed to investigate the relationship between the miRNA profile and metabolic changes over time in women with PCOS. The women with PCOS changed clinically during the 6-yr FU towards a more impaired metabolic (in terms of increasing BMI, insulin levels, HOMA-IR, and levels of C-peptide) and less hyperandrogenic (in terms of decreasing DHEAS, SHBG, total T, free T, and androstenedione). Meanwhile, only a few parameters changed significantly in the control group.

While the connection between Type 2 diabetes (T2D) and PCOS is well established [4,38], the progression in cardiometabolic risk over time in women with PCOS is still being debated. A recent systematic review [39] reports inconsistent findings in longitudinal studies comparing cardiometabolic risk factors (BMI, waist circumference, blood pressure, lipid profile, impaired glucose tolerance, and T2D) in women with PCOS with controls. They conclude that the changes in these risk factors over time are similar in women with PCOS and controls except for the incidence of T2D and IGT. This is in line with our findings, except for the change over time in the w/h ratio that was significant in the control group and not in the PCOS group. This could be caused by the comparatively smaller size of the control group.

In our study, we observed differences in the androgen profile between the control group and the PCOS group, as expected at BL, which persisted until FU. Further, all androgen parameters decreased in both groups, although not all significantly in the control group, diminishing the difference between the control group and the PCOS group. This decrease over time in androgen parameters in women with PCOS is also well described in other studies [40,41].

From BL to FU, thirty miRNAs significantly changed in the group of women with PCOS, while none changed in the control group, and with twenty-two of the thirty miRNAs being significantly (following FDR correction) and consistently decreased in circulation. These measured miRNAs were selected based on previously reported associations between circulating levels and traits of PCOS (Table S1). It is therefore evident that the decreased levels of PCOS-associated circulatory miRNAs follow the decreased androgen load of the PCOS patients during the 6-yr FU. These observations strengthen the notion that PCOS-associated miRNAs could be responsive to changes in circulating androgen levels.

Further, of these thirty miRNAs, three miRNAs were also significantly lower in women with PCOS compared with the control group at BL. A fourth miRNA, miR-103a-3p, was also significantly lower in the PCOS group compared with the control group at BL but did not change over time. The change in these four miRNA levels correlated significantly with the change in clinical parameters in the PCOS group. None of the 96 pre-selected miRNAs were significantly different at FU in the PCOS group compared with the control group, but within the PCOS group, a large proportion had decreased. At FU, the PCOS group had changed towards a more non-PCOS-like phenotype, decreasing androgen parameters, while the control group changed towards a more metabolic phenotype with increased BMI and waist-hip ratio. Further, the pre-selected miRNAs were chosen as PCOS-associated miRNAs. These factors constitute likely explanations of why we did not detect differences in miRNA levels between controls and PCOS at FU.

Several studies link miR-28-3p to various cancers, and this miRNA is thus involved in cell proliferation, migration, and invasion [42]. It is located within intron 6 of the LIM domain containing its preferred translocation partner in the lipoma (LPP) gene, and single nucleotide polymorphisms (SNPs) in the LPP gene have been associated with PCOS in a GWAS study [43]. In our study, we found that miR-28-3p was lower in PCOS women and that the levels of miR-28-3p increased at FU. This is in accordance with a study of T2D subjects. Here, lower BL levels of plasma miR-28-3p were found in incident T2DM subjects compared to non-T2D [44]. Using miR-28-3p together with miR-103, miR-29a-3p, and six other miRNAs could predict the development of T2D [44]. On the other hand, a modest increase of BL miR-28-3p was found in another study in patients who later developed T2D [45]. Regardless of the time point, total T consistently correlated positively with miR-28-3p. It could be speculated if miR-28-3p could contribute to follicular atresia through apoptosis of granulosa cells mediated partly via the PI3K-Akt (protein kinase B) pathway or the apoptosis signaling pathway. One of the predicted targets of miR-28-3p is the transcription factor forkhead box O3 (FoxO3), which can induce pro-apoptotic events in the granulosa cells. Indeed, levels of FoxO3 have been observed to be increased in women with PCOS [46], which matches the lower levels of miR-28-3p at BL observed in this study.

Increased BMI has been associated with increased miR-103-3p levels in subcutaneous adipose tissue biopsies from elderly subjects [47]. Furthermore, miR-103-3p is reported as a modulator of glucose metabolism, and inhibition of miR-103-3p improved insulin sensitivity [48]. We did not observe any correlations between BMI and miR-103-3p, nor were markers related to glucose metabolism associated with miR-103-3p. However, predicted target genes belonging to the fibroblast growth factor (FGF)-signaling pathway was significantly enriched. Several FGFs are involved in either promoting granulosa cell proliferation or atresia in ovarian follicles. Thus, dysregulation hereof, partly mediated by miR-103, could explain phenotypical characteristics of the syndrome that persist despite the fact that the six-year FU level of miR-103 did not change significantly within this time frame.

Upregulation of miR-139-5p lowered blood glucose levels and was able to protect diabetic mice from liver damage by oxidative stress [49]. As an additional layer of complexity, interactions between miRNAs and circular RNAs (circRNAs) can affect the downstream targets of these miRNAs, thus contributing further to the hormonal imbalance. Using expression datasets associated with PCOS, five circRNAs were predicted to have the capabilities of sponging miR-139-5p [50]. Thus lower BL miR-139-5p could be due to an indirect sponging effect of these circRNAs. However, further validation experiments are needed to confirm this hypothesis. Several transcripts targeted by miR-139-5p were associated with three pathways: angiogenesis, cadherin, and Wnt signaling pathways (Figure 4). Interestingly, miR-139-5p is shown to both negatively regulate Wnt signaling [51] and be repressed by the effectors of Wnt signaling, beta-catenin, and TCF4 [52], forming a reciprocal balanced feedback mechanism. Of note, Wnt signaling is important for the maintenance of ovarian folliculogenesis [53].

MiR-376a-3p is predicted to target angiogenesis, de novo pyrimidine synthesis, as well as several types of glutamatergic signaling. Of possible importance, glutamatergic signaling is required for the neuroendocrine feedback of gonadal steroid hormones on GnRH neurons [54]. Pyrimidine synthesis is required during DNA synthesis for cell division, and in corona radiata, cumulus cells from PCOS patients were found to be upregulated [55], consistent with lower circulating levels of miR-376-3p in PCOS patients at BL but not at FU.

ROC analysis for the four miRNAs indicated AUCs of 0.807-0.891, which is comparable to that of free T (Figure 3 and Figure S1), an important benchmark for PCOS-associated miRNAs, and should these be deemed suitable for further biomarker development. Of note, the ROC analyses were performed for each miRNA independently, and it is possible that in the future, several miRNAs may be combined to form biomarker algorithms.

The four miRNAs upregulated during FU In PCOS patients (Table 2), pathways such as initial and antibody triggering of complement, activation of C4 and C2, B-cell receptor regulation, and antimicrobial peptides are depleted for mRNA targets of the upregulated miRNAs, possibly suggesting a relatively lower impact of the upregulated miRNAs. The twenty-six miRNAs down-regulated during FU in PCOS patients in total target mRNAs were significantly enriched in pathways such as membrane trafficking, nervous system development, axon guidance, GnRH receptor pathways, and post-translational modification (Figure 5), suggesting that target gene depression could occur during FU to allow for increased GnRH signaling and, possibly, improved feedback from gonadal steroids [54].

We determined the change in miRNA levels during follow-up in a period where the PCOS phenotype decreased. However, it should also be noted that a number of confounding factors have also been demonstrated to change miRNA levels: Age has been shown to change circulating miRNA levels, and miR-151-5p was decreased during aging, similar to our observations (Table 2) [56], while miR-222-3p was increased during aging, opposite to the direction observed by us [57]. Moreover, metformin was used by some of the PCOS women in our study and has been shown to increase levels of miR-20a-5p [58] and decreased levels of miR-151-3p [59], while we observed significantly decreased levels of miR-20a-5p and miR-151-3p (Table 2). Thus, aging and metformin use change the levels of specific miRNAs in circulation, and it will be important to adjust for such confounders should specific miRNAs be developed as biomarkers in the future.

The strength of this study is the longitudinal design and the ability to track changes in miRNA in women with PCOS over time, which, to our knowledge, has not been conducted before. However, the major limitation of this study is the limited size of the control group, but also generally the drop-out rate from BL to FU. The low number of participating controls was the result of a recruitment issue, but was also caused by the fact that 22.1% of the former participants were excluded from participation in the FU study due to one of the listed criteria (Figure 1). Therefore, we have the most power to detect miRNAs that change over time in the PCOS group, rather than being able to detect differences between controls and women with PCOS. Interpretations of the differences should therefore be performed with this in mind.

In this study, we examined a panel of 96 miRNAs previously associated with PCOS and related phenotypes for their levels in circulation in a 6-yr FU in a small cohort of nine control and 46 PCOS women examined at BL before ART and re-examined 6 yr later. Thirty of these miRNAs were significantly changed, with the majority decreasing their levels during FU, concomitant with PCOS patients experiencing a decrease in androgen status. Four of the miRNAs (miR-28-3p, miR-103-3p, miR-139-5p, and miR-376a-3p) were regulated differently over time in controls and PCOS women, their levels correlating with free T or total T, with pathway analyses indicating enrichment of GnRH and inflammatory pathways.

In conclusion, our data points towards a circulating miRNA profile of PCOS associated miRNAs that overall decrease over a 6-year FU period to mirror the phenotypic changes in PCOS patients, which become less burdened by hyperandrogenism.

## Figures and Tables

**Table 1:** BL and FU characteristics of the study participants. Data are represented as means (SD) or as medians (interquartile range) if not normally distributed. * *p* < 0.05, ** *p* < 0.01, *** *p* < 0.001.

	BL			FU			BL vs. FU	
	Control	PCOS	*p*	Control	PCOS	*p*	Control *p*	PCOS *p*
Age (years)	30.2 (4.9)	28.5(4.2)		36.0 (5.7)	34.3 (4.3)		-	-
Weight (kg)	74.6(13.5)	77.2 (19.9)		79.8 (12.1)	78.2 (17.7)		-	-
Height (cm)	169.8 (3.6)	168.0 (6.8)		170.1 (5.3)	168.1 (6.7)		-	-
BMI (kg/m^2^)	25.6 (4.7)	26.8 (5.5)		27.7 (4.4)	27.7 (6.1)		*	*
Waist-Hip ratio	0.78 (0.1)	0.83 (0.1)	*	0.85 (0.6)	0.84 (0.1)		*	-
Systolic Blood pressure (mmHg)	124.1 (16.1)	122.4 (10.9)		116.2 (18.6)	120.7 (13.5)		-	-
Diastolic Blood pressure (mmHg)	78.9 (14.1)	76.3 (10.3)		72.7 (16.4)	72.3 (11.2)		-	*
**Metabolic Characteristics**								
F-p-Glucose (mmol/L)	5.3 (4.7–6.0)	5.2 (5.0–5.4)		4.7 (4.6–5.2)	4.8 (4.6–5.1)		-	**
F-s-Insulin (pmol/L)	61.8 (44.9–75.9)	45.8 (30.4–104.1)		48.1 (37.0–67.0)	70.0 (37.8–108.9)		-	**
F-s-C-peptide (nmol/L)	0.6 (0.6–0.7)	0.6 (0.4–0.8)		0.6 (0.6–0.7)	0.7 (0.5–1.1)		-	***
HOMA-IR (mU*mmol/L^2^)	2.1 (1.5–2.6)	1.5 (1.0–3.3)		1.5 (1.1–1.9)	1.9 (1.1–3.6)		-	*
HDL cholesterol (mmol/L)	1.5 (1.3–1.6)	1.5 (1.2–1.8)		1.4 (1.3–1.4)	1.4 (1.2–1.6)		-	-
LDL cholesterol (mmol/L)	3.1 (2.6–3.5)	2.6 (2.1–3.1)		2.9 (2.1–3.1)	2.4 (2.1–3.0)		-	-
Total cholesterol (mmol/L)	4.9 (4.3–5.1)	4.5 (4.1–5.0)		4.6 (3.8–4.8)	4.4 (4.0–5.0)		-	-
Triglyceride (mmol/L)	0.7 (0.7–0.8)	0.7 (0.6–1.2)		0.9 (0.8–1.1)	0.8 (0.7–1.5)		-	**
**Androgen Markers**								
DHEAS (Umol/L)	5.29 (1.86)	6.27 (2.24)		4.33 (2.41)	4.81 (2.13)		-	**
SHBG (nmol/L)	71.0 (47.0–75.0)	59.5 (36.0–89.0)		53.0 (32.0–66.0)	46.0 (35.0–82.0)		-	**
Total testosterone (nmol/L)	1.0 (0.8–1.5)	1.9 (1.5–2.6)	***	0.9 (0.4–0.9)	1.4 (0.9–2.0)	**	*	***
Free testosterone (nmol/L)	0.017 (0.013–0.02)	0.032 (0.019–0.05)	**	0.014 (0.010–0.015)	0.023 (0.012–0.05)		-	**
Androstenedione (nmol/L)	4.2 (3.4–5.2)	6.9 (4.7–9.0)	**	2.6 (2.2–3.2)	5.8 (4.2–8.9)	***	***	*
LH/FSH ratio	0.9 (0.6–1.2)	1.7 (1.2–2.4)	***	0.8 (0.8–1.1)	1.5 (1.0–2.2)	*	-	*
Ferriman-Gallwey score	2.0 (2.0–3.0)	5.0 (3.0–9.5)	**	1.00 (0.0–5.0)	6.0 (3.0–9.0)	**	-	-
**Lifestyle characteristics**								
Metformin use (current/previous/no)					9/22/14			
ART outcome successful (yes/no)				7/2	38/8			
Smoking (current, previous, no)				3/2/4	9/11/26			
Alcohol consumption (yes, no)				3/6	10/36			

**Table 2:** Thirty miRNAs change over time in PCOS women. Shown are the mean fold change ± standard deviation as well as the number of women included. Changes in expression levels were evaluated with a paired student *t*-test. *p* values were corrected for multiple testing using Bonferroni corrections. MiRNAs are human miRNAs (*hsa-miR*) unless otherwise specified. Some assays were designed for murine miRNAs, but in these cases, the miRNA was completely homologous. PCOS (FF): Levels in follicular fluid associated with PCOS, PCOS (circulating): Levels in circulation associated with PCOS. NAFLD: Non-alcoholic fatty liver disease.

miRNA	Reported Association	Difference in dCt ± SD	Direction	*n*	*p*	p_adj_
miR-451a	PCOS (circulating)	6.3 ± 3.2	Decreased	34	5.7 × 10^−13^	5.5 × 10^−11^
miR-24-3p	PCOS (FF)	5.8 ± 5.2	Decreased	42	8.4 × 10^−9^	8.1 × 10^−7^
miR-16-5p	PCOS	5.3 ± 3.1	Decreased	39	4.8 × 10^−13^	4.6 × 10^−11^
miR-21-5p	PCOS (circulating)	4.6 ± 3	Decreased	27	2.2 × 10^−8^	2.1 × 10^−6^
miR-142-3p	Dysregulated in PCOS granulosa cells	4.5 ± 3.3	Decreased	24	8.4 × 10^−7^	8.1 × 10^−5^
miR-223-3p	PCOS (circulating	4.5 ± 3.2	Decreased	43	1.4 × 10^−11^	1.3 × 10^−9^
miR-720	PCOS (FF)	4.1 ± 3.2	Decreased	44	1.1 × 10^−10^	1.1 × 10^−8^
miR-19b-3p	PCOS (circulating)	4 ± 3	Decreased	38	7.3 × 10^−10^	7.0 × 10^−8^
miR-151-3p	PCOS (FF)	3.8 ± 2	Decreased	36	2.2 × 10^−13^	2.1 × 10^−11^
miR-342-3p	Circulating levels associated with insulin resistance	3.7 ± 2.7	Decreased	35	2.3 × 10^−9^	2.2 × 10^−7^
miR-146a-5p	PCOS (circulating)	3.6 ± 2.5	Decreased	39	7.4 × 10^−11^	7.1 × 10^−9^
miR-126-3p	PCOS (circulating)	3.4 ± 2.6	Decreased	37	3.6 × 10^−9^	3.4 × 10^−7^
miR-93-5p	PCOS (circulating)	3.2 ± 2.5	Decreased	35	9.9 × 10^−9^	9.5 × 10^−7^
miR-145-5p	PCOS (circulating)	3.2 ± 2.1	Decreased	32	1.5 × 10^−9^	1.5 × 10^−7^
miR-1305	Circulating biomarkers of ovarian cancer	3.1 ± 5.9	Decreased	24	1.6 × 10^−2^	1.0
miR-320a-3p	PCOS (FF)	3.1 ± 1.8	Decreased	42	3.2 × 10^−14^	3.1 × 10^−12^
miR-151-5p	PCOS (FF)	2.7 ± 4.2	Decreased	29	1.8 × 10^−3^	0.17
miR-17-5p	Circulating biomarkers of metabolic syndrome	2.7 ± 2.6	Decreased	41	9.8 × 10^−8^	9.4 × 10^−6^
miR-423-5p	Dysregulated in PCOS granulosa cells	2.5 ± 1.8	Decreased	33	4.0 × 10^−9^	3.8 × 10^−7^
miR-139-5p	PCOS (circulating)	2.5 ± 2.0	Decreased	30	2.4 × 10^−7^	2.3 × 10^−5^
miR-28-3p	Granulosa cell proliferation	2.3 ± 1.8	Decreased	25	7.9 × 10^−7^	7.6 × 10^−5^
miR-30b-5p	Associated with NAFLD	2.2 ± 2.4	Decreased	33	6.8 × 10^−6^	6.6 × 10^−4^
miR-30c-5p	PCOS (circulating)	2.2 ± 2.3	Decreased	37	1.2 × 10^−6^	1.2 × 10^−4^
miR-20a-5p	PCOS (circulating)	1.9 ± 2.9	Decreased	33	5.4 × 10^−4^	0.052
miR-222-3p	PCOS (circulating)	1.8 ± 2.5	Decreased	35	2.1 × 10^−4^	0.020
miR-221-3p	Associated with inflammation	1.7 ± 2.5	Decreased	26	1.4 × 10^−3^	0.130
miR-1233-3p	Circulating biomarkers of ovarian cancer	5.5 ± 7.1	Increased	28	3.2 × 10^−4^	0.031
miR-518f-3p	PCOS (FF)	6.6 ± 6.3	Increased	34	7.9 × 10^−7^	7.6 × 10^−5^
miR-520c-3p	PCOS (FF)	8.4 ± 8.1	Increased	30	3.7 × 10^−6^	3.6 × 10^−4^
miR-618	Not previously published	10.3 ± 7.5	Increased	29	5.1 × 10^−8^	4.9 × 10^−6^

**Table 3:** Correlation between clinical and biochemical markers with the four selected miRNA within the PCOS women. The individual time points (BL or FU) are kept separate. Spearman rho is displayed, as well as the number of women included. P_adj_ (two-tailed, partial correlations): correlations between the selected variable controlling for age and BMI at the respective time points. N: number of PCOS women included in the analysis.

Time Point	Clinical/Biochemical Marker	MiRNA	N	Spearman Rho (ρ)	*p*	p_adj._
BL	Total T	miR-139-5p	34	0.50	0.002	**0.013**
		miR-28-3p	31	0.37	0.043	0.090
	Free T	miR-139-5p	34	0.47	0.005	0.051
FU	Waist-hip ratio	miR-376a-3p	23	0.43	0.042	**0.010**
	Total T	miR-28-3p	35	0.40	0.016	**0.031**

## Data Availability

All data reported herein are displayed in the article figures, tables, and supplementary material.

## Supplementary Materials

The following supporting information can be downloaded at: https://www.mdpi.com/article/10.3390/cells12070983/s1, Table S1: List of microRNAs, Taqman, miRNA mature sequences tested on the 96-miR custom card and phenotype associations previously reported. Table S2: Change in miRNA levels in women without PCOS from BL to FU. Figure S1: ROC curve of free T in which age and BMI at BL was included in the logistic regression model. Figure S2: Correlations between clinical and biochemical variables and the selected miRNAs, at either BL or FU, measured in women with PCOS.
