# Supplementary material for: Circulating miRNAs in Women with Polycystic Ovary Syndrome: A Longitudinal Cohort Study
Source: Cells. 2023 Mar 23;12(7):983. doi: 10.3390/cells12070983 (PMC10093401; doi:10.3390/cells12070983)
Supplement: Supplementary file 1 [file cells-12-00983-s001.zip › cells-2149142-supplementary.pdf]

cells

MDPI

Article

Supplementary Materials: Circulating miRNAs in Women with Polycystic Ovary Syndrome: A Longitudinal Cohort Study

Pernille B. Udesen [1,†], Anja E. Sørensen [2,†], Rikke Svendsen [2], Nanna L. S. Frisk [2], Anne L. Hess [3], Mubeena Aziz [4], Marie Louise M. Wissing [2], Anne Lis M. Englund [1] and Louise T. Dalgaard [2,*]

Supplementary Table S1: List of microRNAs, Taqman, miRNA mature sequences tested on the 96-miR custom card and phenotype associations previously reported

Mature miRNA	TaqMan ID	Sequence	Phenotype association	Pubmed reference ID
ath-miR-159a	338	uuuggauugaagggagcucua	-	
cel-miR-39-3p	200	ucaccggguguaaaucagcuug	-	
dme-miR-7-5p	268	uggaagacuagugauuuuguugu	-	
U6	1973	-	-	
miR-133a-3p	2246	uuuggucccсuucaaccagcug	PCOS (FF)	PMID: 23666971
miR-625-3p	2431	gacuauagaacuuucccccuca	Associated with cholesterol levels	PMID: 24784704
mmu-miR-140-5p	1187	cagugguuuuacccuaugguag	Associated with granulosa cell proliferation	PMID: 32024547
miR-1225-3p	2766	ugagccccugugccgcccccag	Associated with inflammation	PMID: 29496996
miR-519b-3p	2384	aaagugcauccuuuuagagguu	Associated with inflammation	PMID: 32146774
miR-221-3p	524	agcuacauugucugcuggguuuc	Associated with inflammation	PMID: 35120179
miR-107	443	agcagcauuguacagggcuauca	Associated with insulin resistance	PMID: 21654750
miR-664a-3p	2897	uauucauuuauccccagccuaca	Associated with insulin resistance	PMID: 29495974
miR-363-3p	1271	aauugcacgguauccaucugua	Associated with insulin resistance	PMID: 32104036
miR-886-3p	2194	cgcgggugcuuacugacccuu	Associated with metabolic syndrome	PMID: 34294131
miR-122-5p	2245	uggagugugacaaugguguuug	Associated with metabolic syndrome and Type 2 diabetes	PMID: 27899485
miR-30b-5p	602	uguaaacauccuacacucagcu	Associated with NAFLD	PMID: 28119530

miR-190b	2263	ugauauguuugauauuggguu	Associated with NAFLD	PMID: 29575055
miR-629-5p	2436	uggguuuacguugggagaacu	Biomarker of ovarian cancer	PMID: 28972400
miR-193b-3p	2367	aacuggcccucaaagucccgcu	Circulating biomarker for impaired glucose tolerance in PCOS	PMID: 26860517
miR-194-5p	493	uguaacagcaacuccaugugga	Circulating biomarker for impaired glucose tolerance in PCOS	PMID: 26860517
miR-19b-3p	396	ugugcaaauccaugcaaaacuga	Circulating biomarker for PCOS	PMID: 32411089
miR-17-5p	2308	caaagugcuuacagugcagguag	Circulating biomarker of metabolic syndrome	PMID: 23032062
miR-195-5p	494	uagcagcacagaaauauuggc	Circulating biomarker of metabolic syndrome	PMID: 23032062
miR-324-3p	2161	acugccccaggugcugcugg	Circulating biomarker of metabolic syndrome	PMID: 32353552
miR-148a-3p	470	ucagugcacuacagaacuuugu	Circulating biomarker of metabolic syndrome	PMID: 34954485
miR-215-5p	518	augaccuaugaauugacagac	Circulating biomarker of obesity	PMID: 30651891
miR-532-5p	1518	caugccuugaguguaggaccgu	Circulating biomarker of ovarian cancer	PMID: 26873729
miR-376a-3p	565	aucauagaggaaaauccacgu	Circulating biomarker of ovarian cancer	PMID: 27979415
miR-518b	1156	caaagcgcuccccuuuagaggu	Circulating biomarker of ovarian cancer	PMID: 32083575
miR-1305	2867	uuuucaacucuaaugggagaga	Circulating biomarker of ovarian cancer	PMID: 32854743
miR-766-3p	1986	acuccagccccacagccucagc	Circulating biomarker of ovarian cancer	PMID: 32854743
miR-1233-3p	2768	ugagcccuguccucccgcag	Circulating biomarker of ovarian cancer	PMID: 34899827
miR-222-3p	2276	agcuacaucuggcuacugggu	PCOS (circulating)	PMID: 24802714
miR-30c-5p	419	uguaaacauccuacacucucagc	PCOS (circulating)	PMID: 24802714
miR-223-3p	2295	ugucaguuugucaaauacccca	PCOS (circulating)	PMID: 26582398

mmu-miR-93-5p	1090	caaagugcuguucgugcagguag	PCOS (circulating)	PMID: 26582398
miR-4522	462414_mat	ugacucugccuguaggccggu	PCOS (circulating)	PMID: 27677182
miR-6767-5p	466470_mat	ucgcagacagggacacauggaga	PCOS (circulating)	PMID: 27677182
miR-23a-3p	399	aucacauugccagggauuucc	PCOS (circulating)	PMID: 28193283
miR-23b-3p	400	aucacauugccagggauuacc	PCOS (circulating)	PMID: 28193283
miR-9-5p	583	ucuuugguuaucuagcuguauga	PCOS (circulating)	PMID: 30794820
mmu-miR-451a	1141	aaaccguuaccauuacugaguu	PCOS (circulating)	PMID: 31730174
miR-139-5p	2289	ucuacagugcacgugucuccagu	PCOS (circulating)	PMID: 31737638
miR-143-3p	2249	ugagaugaagcacuguagcuc	PCOS (circulating)	PMID: 31737638
miR-21-5p	397	uagcuuaucagacugauguuga	PCOS (circulating)	PMID: 31737638
miR-34a-5p	426	uggcagugucuuagcugguugu	PCOS (circulating)	PMID: 31737638
miR-34b-3p	2102	caaucacuaacuccacugccau	PCOS (circulating)	PMID: 31737638
miR-361-5p	554	uuaucagaaucuccaggggguac	PCOS (circulating)	PMID: 31737638
miR-433-3p	1028	aucaugaugggcuccucggugu	PCOS (circulating)	PMID: 31737638
miR-485-3p	1277	gucauacacggcucuccucucu	PCOS (circulating)	PMID: 31737638
miR-572	1614	guccgcucggcgguggccca	PCOS (circulating)	PMID: 31737638
miR-27b-3p	409	uucacaguggcuaaguucugc	PCOS (circulating)	PMID: 32848854

miR-106a-5p	2169	aaaagugcuuacagugcagguag	PCOS (circulating)	PMID: 33041053
miR-126-3p	2228	ucguaccgugaguaauaaugcg	PCOS (circulating)	PMID: 33041053
miR-146a-5p	468	ugagaacugaauuccaugggeuu	PCOS (circulating)	PMID: 33041053
miR-18b-5p	2217	uaaggugcaucuagugcaguuag	PCOS (circulating)	PMID: 33041053
miR-20a-5p	580	uaaagugcuuauagugcagguag	PCOS (circulating)	PMID: 33041053
miR-16-5p	391	uagcagcacguaaauauuggcg	PCOS (circulating)	PMID: 33148068
miR-145-5p	2278	guccaguuuucccaggaaucccu	PCOS (circulating)	PMID: 33763302
miR-192-5p	491	cugaccuaugaauugacagcc	PCOS (circulating)	PMID: 33763302
miR-103a-3p	439	agcagcauuguacagggcuauga	PCOS (circulating)	PMID: 34403358
miR-342-3p	2260	ucucacacagaaaucgcacccgu	Insulin resistance (circulating)	PMID: 24784704
miR-331-3p	545	gccccugggccuauccuagaa	Fatty acids (circulating)	PMID: 24784704
miR-330-3p	544	gcaaagcacacggccugcagaga	Circulating levels regulated by fatty acids	PMID: 26092372
miR-769-5p	1998	ugagaccucuggguucugagcu	Circulating levels regulated by fatty acids	PMID: 26092372
miR-155-5p	2623	uuaaugcuaaucgugauaggggu	Circulating biomarker of metabolic syndrome and inflammation	PMID: 26421388
miR-130a-3p	454	cagugcaaugaugaaagggcau	Controls theca cell androgen production	PMID: 31184707
miR-142-3p	464	uguaguguuuccuacuuuaugga	Dysregulated in PCOS granulosa cells	PMID: 30865275

miR-423-5p	2340	ugaggggcagagagcgagacuuu	Dysregulated in PCOS granulosa cells	PMID: 30865275
miR-135a-5p	460	uauggcuuuuuauuccuauguga	Dysregulated in PCOS granulosa cells	PMID: 31173821
miR-429	1024	uaauacugucugguaaaaccgu	Dysregulated in PCOS granulosa cells	PMID: 32759991
miR-224-5p	2099	caagucacuagugguuccguu	Granulosa cell proliferation	PMID: 20118412
miR-28-3p	2446	cacuagauugugagcuccugga	Granulosa cell proliferation	PMID: 31322191
miR-200b-3p	2251	uaauacugccugguaaugauga	Granulosa cell proliferation	PMID: 31421682
let-7d-5p	2283	agagguaguagguugcauaguu	Granulosa cell proliferation	PMID: 34246727
miR-448	1029	uugcauauguaggaugucccau	Involved in ovarian cancer growth	PMID: 26103953
miR-520c-3p	2400	aaagugcuuccuuuuagagggu	PCOS (FF)	PMID: 23666971
miR-383-5p	573	agaucagaaggugauuguggcu	PCOS (FF)	PMID: 24828505
miR-132-3p	457	uaacagucuacagccauggucg	PCOS (FF)	PMID: 26771704
miR-151-3p	2254	cuagacugaagcuccuugagg	PCOS (FF)	PMID: 26771704
miR-151-5p	2642	ucgaggagcucacagucuagu	PCOS (FF)	PMID: 26771704
miR-24-3p	402	uggcucaguucagcaggaacag	PCOS (FF)	PMID: 26771704
miR-29a-3p	2112	uagcaccaucugaaaucgguua	PCOS (FF)	PMID: 26771704
miR-320a	2277	aaaagcuggguugagagggcga	PCOS (FF)	PMID: 26771704
miR-518f-3p	2388	gaaagcgcuucucuuuagagg	PCOS (FF)	PMID: 26771704
miR-574-3p	2349	cacgcucaugcacacacccaca	PCOS (FF)	PMID: 26771704
miR-720	2895	ucucgcuggggccucca	PCOS (FF)	PMID: 26771704
miR-127-3p	452	ucggauccgucugagcuuggcu	PCOS (FF)	PMID: 31705013
miR-199b-5p	500	cccaguguuuagacuaucuguuc	PCOS (FF)	PMID: 31705013

miR-484	1821	ucaggcucaguccccucccgau	Possible reference miR	PMID: 31737638
miR-1267	2885	ccuguugaaguguaaucccca	Preliminary inhouse data	-
miR-618	1593	aaacucuacuuguccuucugagu	Preliminary inhouse data	-
miR-636	2088	ugugcuugcucgucccgcccgca	Preliminary inhouse data	-
miR-138-5p	2284	agcugguguugugaaucaggccg	Regulated by estradiol	PMID: 24205079
mmu-miR-96-5p	186	uuuggcacuagcacauuuuugcu	Regulated by estradiol	PMID: 24205079
miR-10b-5p	2218	uacccuguagaaccgaauuugug	Regulated by estradiol	PMID: 33982889

MicroRNAs are human microRNAs (*hsa-miR*) unless otherwise specified. Some assays were designed for murine miRNAs, but in these cases the miRNA was completely homologous. PCOS (FF): Levels in follicular fluid associated with PCOS, PCOS (circulating): Levels in circulation associated with PCOS. NAFLD: Non-alcoholic fatty liver disease.

Supplementary Table S2: Change in miRNA levels in women without PCOS from BL to FU.

miRNA Control	Reported association	Difference in dCt (±SD)	Direction	n	p	p_{adj}
miR-451a	PCOS (circulating)	6.5 ± 3.5	Decreased	7	0.003	0.29
miR-16-5p	PCOS (circulating)	5.1 ± 4.7	Decreased	6	0.044	1.0
miR-223-3p	PCOS (circulating)	4.4 ± 3.8	Decreased	8	0.013	1.0
miR-532-5p	Ovarian cancer (circulating)	4.4 ± 1.1	Decreased	3	0.021	1.0
miR-1305	Ovarian cancer (circulating)	4.1 ± 2.1	Decreased	4	0.03	1.0
miR-24-3p	PCOS (FF)	4.1 ± 3.9	Decreased	7	0.032	1.0
miR-342-3p	Circulating levels associated with insulin resistance	3.8 ± 3.1	Decreased	7	0.017	1.0
miR-720	PCOS (FF)	3.4 ± 2.8	Decreased	8	0.01	0.96
miR-145-5p	PCOS (circulating)	3.3 ± 1.8	Decreased	7	0.003	0.29
miR-127-3p	PCOS (FF)	3.1 ± 0.7	Decreased	3	0.017	1.0
miR-151-3p	PCOS (FF)	3.1 ± 2	Decreased	7	0.006	0.58
miR-146a-5p	PCOS (circulating)	3 ± 2.7	Decreased	6	0.042	1.0
miR-130a-3p	Controls theca cell androgen production	3 ± 0.4	Decreased	3	0.005	0.48
miR-320a-3p	PCOS (FF)	2.6 ± 1	Decreased	7	0.001	0.10
miR-423-5p	Dysregulated in PCOS granulosa cells	2.3 ± 0.8	Decreased	4	0.01	0.96
miR-886-3p	Associated with metabolic syndrome	1.8 ± 1	Decreased	5	0.018	1.0
miR-518f-3p	PCOS (FF)	7.4 ± 6.9	Increased	6	0.047	1.0
miR-520c-3p	PCOS (FF)	10.6 ± 7.5	Increased	6	0.018	1.0
miR-618	-	11.1 ± 9	Increased	6	0.03	1.0
miR-636	-	11.7 ± 7	Increased	4	0.044	1.0
miR-518b	Ovarian cancer (circulating)	15.9 ± 8.9	Increased	5	0.017	1.0

Supplementary Table S2: Changes in miRNA levels from BL to FU in control women. Shown are the mean fold change ± standard deviation as well as the number of women included. Changes in expression levels were evaluated with a paired student t-test. *) also reported in PCOS women in the current study. P values were corrected for multiple testing using Bonferroni corrections. PCOS (FF): Levels in follicular fluid associated with PCOS, PCOS (circulating): Levels in circulation associated with PCOS. Ovarian cancer (circulating): Circulating biomarker of ovarian cancer.

Supplementary Figure S1: ROC curve of free T

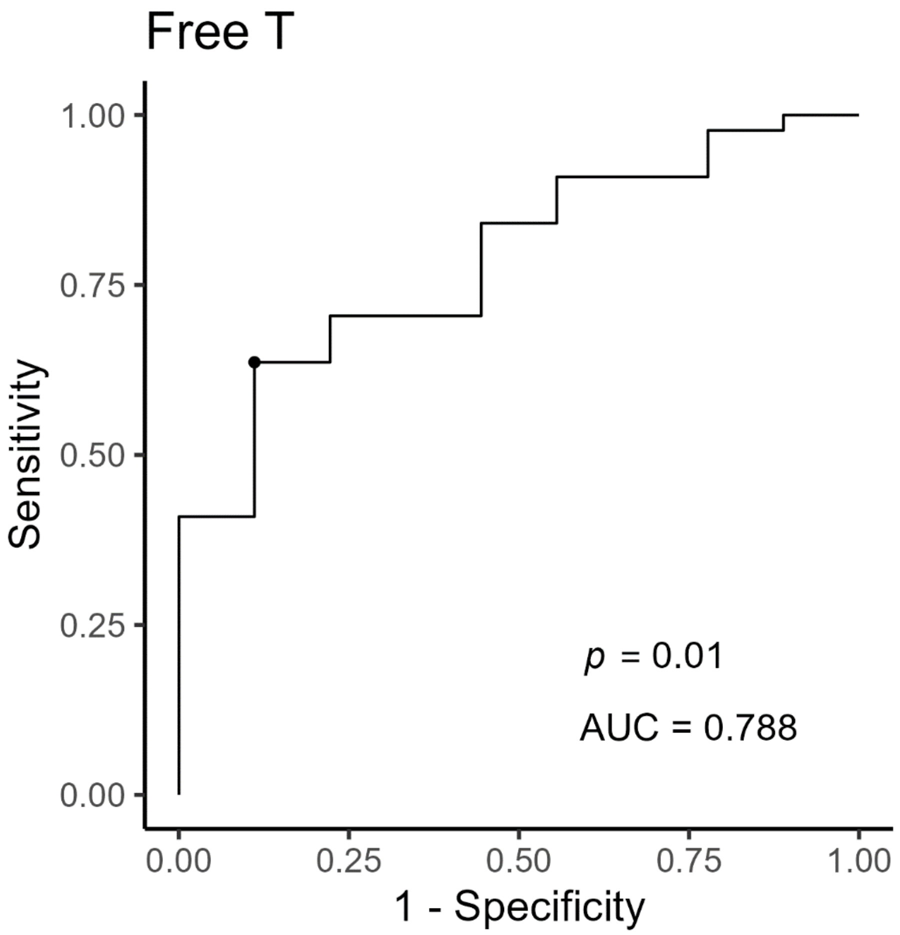

Supplementary Figure S1: ROC curve of free T in which age and BMI at BL was included in the logistic regression model. n = 53, sensitivity = 63% while specificity = 89%.

Supplementary Figure S2: Correlations between clinical and biochemical variables and miRNAs for women with PCOS, at BL or FU.

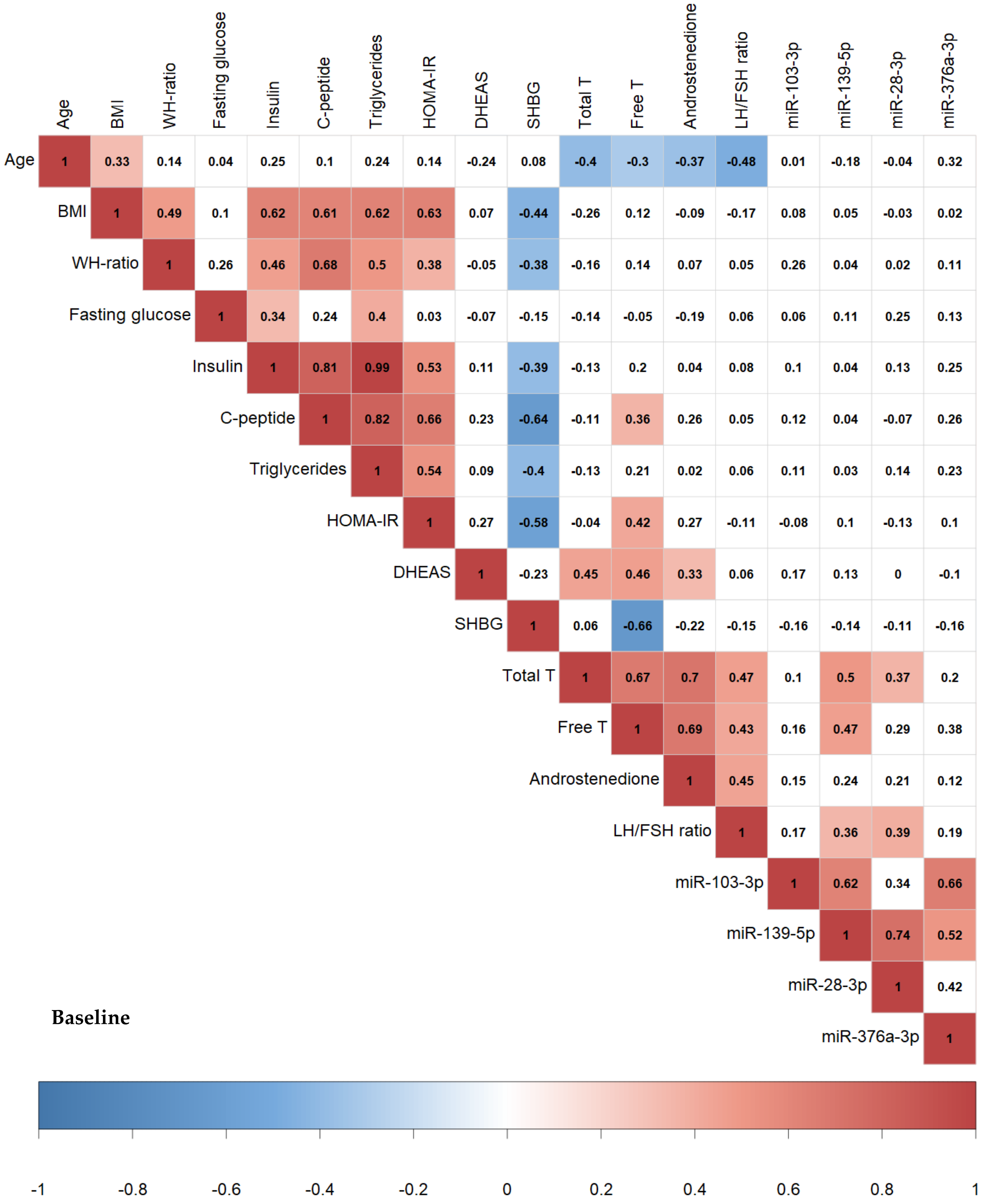

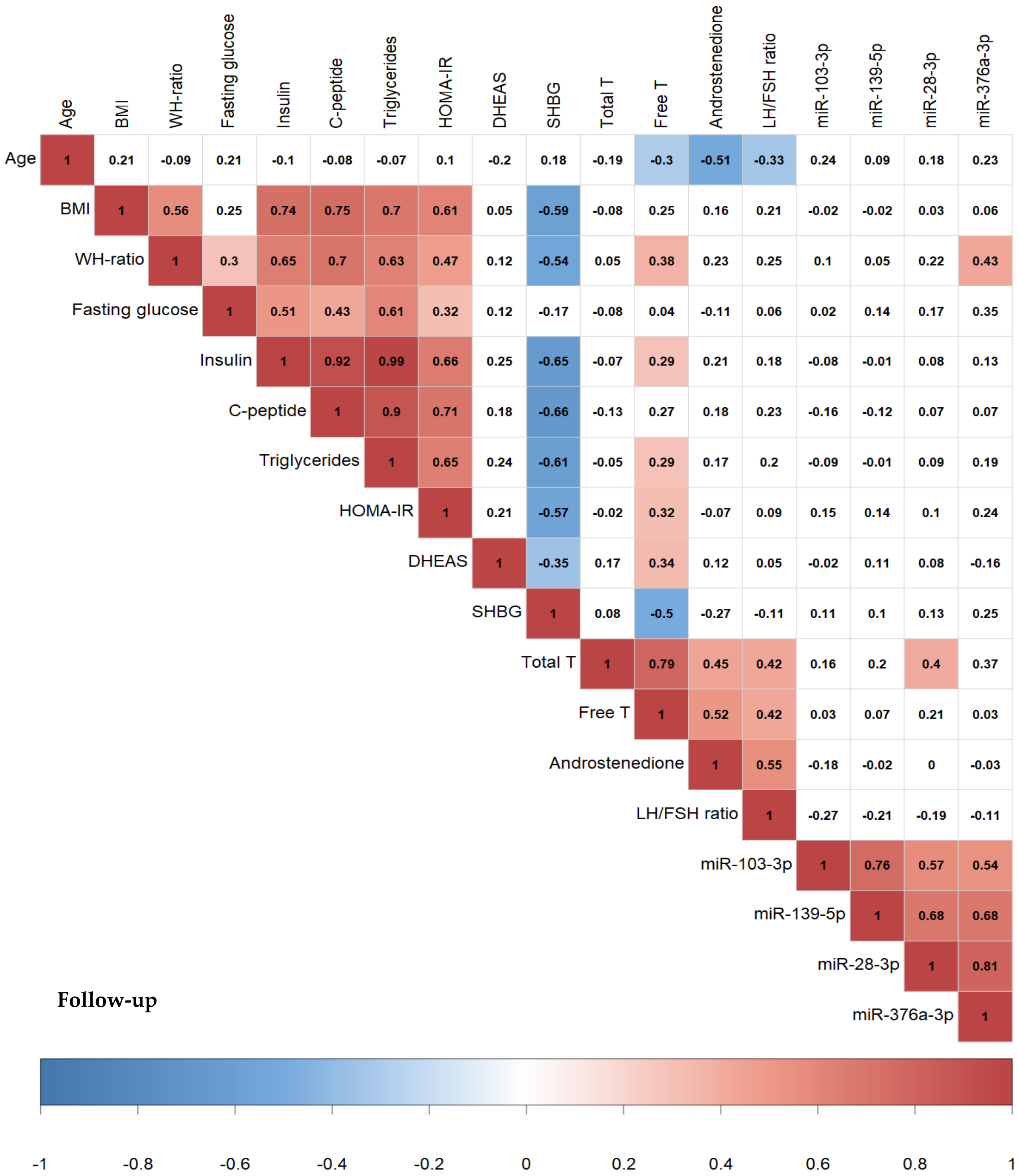

Supplementary Figure S2: Correlations between clinical and biochemical variables and the selected miRNAs, at either BL or FU, measured in women with PCOS. Colors represent significant ($p \leq 0.05$) correlations by Spearman rho with color intensity ranging from -1 to +1.
